# The Nursing Supplement

**Published:** 1889-12-28

**Authors:** 


Being the Extra Nursing Supplement of "The Hospital" Newspaper.

All Contributions for this Supplement should be addressed to the Editor, The Hospital, 139-140, Salisbury Court, Fleet Street, London, E.C., and should have the word "Nursing" plainly written in left-hand top corner of the envelope.

### En Passant.

LOOKING BACK.—It is impossible, when we stand on the threshold of a new year, not to cast one backward glance into those months which have wrought such changes to many of our lives. Several of the brightest and best spirits in the nursing world have last year won their rest, and we shall see them here face to face no more. Only a few of the numerous deaths on duty have been noted in our pages, but even these few show that a woman takes her life in her hand when she steps into the ranks of those who battle against death and disease. Earthly honours are not for these women ; they pass away and their names fade from memory. But in the great march of progress, in the ever-widening sympathy between rich and poor, in the ever-decreasing power of pain and increase of happiness to all men, their spirit yet lives and works. Their example fires others, and in ever-broadening circles their thoughts and deeds are carried out by those who come after them, and the end of their labours no man can foretell. Only we who look forward fancy we can see in the far future a time of peace and joy, such as this world has never yet known ; a time of universal sympathy and good-will which can only be founded on the lives of these—the martyrs of modern days.

"They climbed the steep ascent to heaven,  
Through peril, toil, and pain ;  
O God, to us may grace be given  
To follow in their train."

GROWING OLD.—Some time ago we had numerous letters from nurses who objected to being called unfit for work at 50 or 60. Unluckily, most nurses of that age are unfit for work in hospital wards, but other branches of the art are open to them. Every institution likes to have a few women of mature age on its books, for often a telegram comes "Don't send a young girl." Then again, if pride is not in the way, the hospital nurse who wants a quiet time in her old age and a good salary, can frequently obtain both by becoming head nurse in a gentleman's family. Her duties there are both light and pleasant if she has a girl under her to do the cleaning.

DISTRICT NURSING.—The Sick Nursing and Mother's Help Society of Stalybridge works on somewhat different lines to most district nursing societies. There are two nurses: Miss Twamley, who trained at Crumpsall Infirmary, and whose services are free to the sick poor ; and Mrs. Farrand, certificated midwife, and Radford medallist of St. Mary's Hospital, Manchester, whose fee is 7s. 6d., including nourishment, bed linen, and baby clothes for a fortnight, or 15s. to those requiring her services only. From June, 1888, to June, 1889, Nurse Twamley attended 130 patients, and Nurse Farrand 70 patients. There have been several enquiries for a night nurse, and the funds of the society are fairly flourishing ; a few more subscribers would admit of one being added to the staff. A large number of articles are kept at the home and lent to those who require them at charges varying from 3d. to 9d. per week. The list includes air-cushions, hot-water bottles, sponges, galvanic machine, Bath chair, crutches, and many more useful articles. The income of the society last year was £160, including £26 received in fees ; the expenditure was £152, including £65, Nurse Twamley's salary, £54, Nurse Farrand's salary.

SECESSION TO ROME.—Miss Pringle, matron of St. Thomas's Hospital, has joined the Roman Catholic Church, and sent in her resignation to the hospital committee. This is said to be the third secession to Rome amongst hospital matrons this year. Is the statement true?

BOLTON WORKHOUSE SCANDAL.—On December 5, Dr. Marsh, the medical officer who was suspended, tendered an apology to the Board of Guardians, and stated his belief that the letters handed him by the late Nurse Morris were forgeries. The apology was declined as unsatisfactory. The meeting was most excited.

RURAL NURSING ASSOCIATION.—This association has been founded to promote trained nursing and midwifery in country districts. The secretary is Mrs. Elizabeth Malleson, Dixton, Winchcombe, Cheltenham, and owing to her efforts a nurse has been started at Kemerton and Gotherington. The association receives subscriptions, and grants aid to districts where a nurse is needed, but funds are short; it encourages lectures on nursing, and discourages uncertificated midwives and nurses. Amongst those who have signified their approval of the scheme are Miss Atkinson, of York-road, Mrs. Dacre Craven, Dr. James H. Aveling, Mrs. Garrett Anderson, and others.

NURSES' HOME AT NEWCASTLE.—This home at 11, Ellison-place is just beginning to pay its way. Nurses are often very hard on institutions, accusing them of under paying their staff, but Miss Emery, the matron of the Newcastle home, says her experience goes to prove that the expenses of maintaining a home are far greater than the nurses imagine. At Newcastle no nurse is taken on the staff unless she produces satisfactory evidence of having trained in a hospital of not less than 100 beds ; strict investigation is made as to her moral character. The nurses are paid as good a salary as the funds of the institution admit; they are cared for in sickness, and are given four weeks' clear holiday in the year. As soon as the committee can afford it they are going to help the nurses to join the Pension Fund. Here, then, is an institution which seems to be reasonably and fairly managed.

CARE OF THE HANDS.—A nurse is often troubled to keep her hands nice and clean ; especially in cold weather, when the skin is liable to get rough or to chap. The hands should be washed as little as possible in warm water ; every night before going to bed warm water may be safely used, but in the morning, and during the daytime, when passing in and out of doors, or through draughty passages, only cold water should be used. The nails must be kept short, and the nail-brush only taken to them when absolutely necessary. Olive-oil or vinegar should be used to remove the black sticky marks of strapping, and a little bicarbonate of soda in water will remove plaster of Paris. After laying out the dead or attending to a body which has been the subject of a post-mortem, a nurse should wash her hands in cold water and use a nail-brush, then soak the hands for a few minutes in weak carbolic, and finally put a little scent on them to remove any disagreeable odour. The constant application of vaseline or other ointments is to be deprecated unless the hands have already chapped and preventive measures are useless. Thoroughly drying the hands after washing generally prevents both roughness and chapping.

### Old Jeremy's Visitor.

"Matilda !"

"I'm a-comin', I'm a-comin'."

"So is Christmas," growled old Mr. Jeremy Crossly.

"That it be, sir ; and very nigh, too," answered Matilda, his old housekeeper, and indeed nurse, entering the room with broad, good-humoured smiles ; "which it's only a matter of a few days now, sir ; and I've had it on my mind to speak to you about ——"

"There, that will do !" snapped Mr. Jeremy. "You know well, Matilda, that I never keep up such ridiculous customs. Bring my supper at once."

"Yes, sir," returned Matilda, disappointedly, but she might have known better than to suppose her old master would enter into any plans for keeping up Christmas.

Mr. Jeremy was a very old man ; very rich, and very lonely. He lived in the old-fashioned house—large and roomy—to which he had brought his young bride so long ago, and whose wide staircases and spacious rooms had once heard the patter of the little feet that belonged to his only child. Mr. Jeremy had been an old man when he married, but his heart was young enough to be nearly broken when he lost his fair young wife. Little Grace, her mother's namesake, grew up in such a dull, saddened home—her mother lost and her father frozen up with sorrow—that when she became a great girl, and went out a little into the world, she was so ignorant of what genuine true love was that she grasped the first offered her, and that, as it happened, was only a sham. She married an adventurer who knew, like everybody else, of old Jeremy's wealth.

"Why," the folk would say, "if he chose, old Jeremy could carpet every room in the old house with golden sovereigns, that is, if he'd choose to draw all his money out of the bank" ; and the little boys would crowd round to listen with wide eyes, and so it got about that old Jeremy was a miser. Whether that were true or not, he was a miserable old body, for when Grace married the adventurer he cast her out of the old home. Nobody knew what had become of her, and Matilda often threw her apron over her head to have a good cry about her young mistress. Things had gone on in this way for a long time, and another Christmas was very near, but it seemed to make no difference to old Jeremy, his heart was as cold and hard as his gold. Certainly, he grew feebler, and was not able to leave the huge armchair set close to the blazing fire all day.

"What did you mean just now, Matilda ?" the old man asked, peering suspiciously at the housekeeper when she set his supper-tray before him. "What was on your mind ? If it were to ask for a rise in your wages it's no good, once for all."

"Well, I'm sure, sir !" Matilda tossed her head with its ancient black cap and blue ribbons ; "that's the last thing you need expect of me. No, sir. What I'd a mind to speak about, sir, was, wouldn't it be a little change for you to see a friend come Christmas ? It 'ud do 'ee good, sir," she ended persuasively.

"What ! A friend ! I haven't any friends. They're all thieves, all sharks !"

"Well, sir, if I might make so bold, Christmas ain't just the time for sayin' so about one's fellow critters. It 'ud be a sight better if you'd pray the Almighty to send the Christ-child to visit your desolate old heart, sir, and melt it." And Matilda left the room, softly shutting the door.

"What could Matilda mean ?" old Jeremy asked himself as he ate his supper. Then, after, as he dozed in the fire-light, he seemed to see a faraway vision of the Christ-child. It was standing a long way off, as if waiting to be beckoned. A storm of wind and rain beat down upon the little fair head ; when the old man awoke with a start he actually felt sorry to think of the child out in the bleak cold. Somehow, that vision did not fade out of his mind for days. Matilda could not quite make her master out. Indeed, Matilda herself was not very easy to make out. The old woman was very busy in her kitchen, making and baking most wonderful cakes, pies, and puddings. The question was, who would eat these good things. Not old Jeremy, certainly.

It was Christmas Eve, and outside there reigned a black frost. Indoors, in the warm old house there were rich, savoury odours stealing about just as if Christmas were going to be kept after all. Mr. Jeremy had been dozing all day in his chair ; Fitz, the terrier, dozed also in front of the fire. The winter afternoon soon closed in, and then the old man fell sound asleep. Again the Christ-child came into his dreams. This time, nearer—and old Jeremy could see it shiver in the frosty air—a little unsheltered child !

"Long ago, surely," dreamed old Jeremy, "I, too, owned a little child. 'Our Grace,' we called her—Grace and I. The two Graces ! And I have lost both !" Then old Jeremy fell awake, and saw a wonderful thing. The Christ-child had got into the warm room ; it was standing on the hearth, its morsels of hands stretched out to the blazing flames, and its blue eyes fixed solemnly on Fitz. Old Jeremy stared silently. What had Matilda said that day ? ". . . . pray the Almighty that the Christ-child should visit your desolate heart." He did not know that he had prayed ; but the prayer seemed to be answered, for there stood the fair little child warming itself.

"Who are you ?" at length quavered the old man.

"I'm little Grace," was the unexpected answer, in a sweet, childish treble.

"Little Grace ! Then, you never grew up, and went away ? It was a mistake ; you're our little Grace !"

"No ;" said the mite, shaking its curls, "I'm mother's little Grace," and she put her arms round Fitz's neck, nearly strangling him, but, oddly enough, Fitz seemed to like it.

"Father ! Dear old dad !" a timid voice came from out of the shadows, and the flames fitfully lighted up a wistful, pale face.

"*That's* 'our Grace,' !" old Jeremy nearly sprang round in his chair, "that's our girl's voice. Come, daughter, come closer ! " And the figure stole swiftly out of the gloom into the firelight.

By-and-by, when Matilda entered the room with lights her good old heart fairly leaped. Seated on the arm of her father's chair, smoothing his wrinkled face, was "our Grace," back in her old place, while at their feet was her Grace—the third of the Graces—whose little fair head leant fearlessly against old Jeremy's knee. Matilda, setting down the lamps, had a struggle to prevent herself flinging her apron over her head for her favourite diversion—a good cry.

"We are going to keep Christmas, Matilda," said her master. "I and the Graces ; so, late as it is, you must do what you can to prepare."

"Very good, sir," meekly replied Matilda, refraining from adding that her preparations were already made. But could that be the old master who spoke ? His face was quite changed, the frozen look was melting already.

"You prayed," went on old Jeremy, "that I might have a visitor. So I have. One of Christ's little ones—and she has brought me my lost daughter."

Old Jeremy had had a visitor, indeed. The real Christ-child had come into his dried-up heart bearing the message : "Good-will towards men." Yes, they kept Christmas in the old house, kept it with thankful hearts, and Matilda, when her tired head lay on its pillow that night, cried and laughed by turns to think how her simple plot had succeeded. For it was the good old soul herself who had found out "our Grace" and her Grace, deserted, desolate, and nigh starving; who had determined to bring about a reconciliation between them and the rich old man into whose pitifully lonely life—late eventide though it was—the light had now come.

### Keeping Christmas.

THE theatricals at Charing Cross were excellent, the acting in the comedy called "The Dowager" being far above the average in amateur entertainments. It was pleasant to see the patients being carried down to front places, and supplied with refreshment in the interval. Three nights were devoted to these theatricals, and on two nights the wards were on view to the visitors, and were lit with fairy lamps. Sir Guyer and Lady Hunter were amongst the audience and their daughter was amongst the performers.

At Kensington Infirmary a delightful concert was given on December 19th, the nurses taking a prominent part in the performance.

The annual concert at Marylebone Infirmary was very good, but there the nurses acted as audience only.

At the London Hospital, on December 20th, a soireé was given by Mr. and Mrs. Carr-Gomm and the Muir family, and a clever ventriloquist helped to amuse the large company assembled. All the nurses and sisters of the hospital were present during some part of the evening, and there were a few visitors in uniform from St. John's House, St. Bartholomew's, and other institutions.

At the Seamen's Hospital the Christmas dinner was a veritable feast for all. The nurses will have a social evening on the 2nd January, commencing at 7.30 p.m., and at the same hour on the 3rd the petty officers will have a smoking concert. On the 6th, at 5 p.m., a Christmas tree will be provided for the few children who during the year found their way, by accident or other urgent causes, into the wards of the Dreadnought Hospital. On the 7th of January the patients will be entertained at a concert.

### Registration.

IT will be seen that Miss Heanley last week made a sensible suggestion in regard to nurses who have been trained at hospitals where there are neither lectures nor examinations. The Committee of the Nurse Training Schools will no doubt consider her proposals in a friendly spirit, and now the memorial of the British Nurses' Association has been made public in these columns they may find themselves in a position to express the views of those who are opposed to the aims of this association. Meanwhile, every certificated nurse will do well to rest content with her certificate, or she may lose caste by having her name inscribed on the common register, to quote the words of the organ of the British Nurses' Association, "amongst hundreds of semi-trained or altogether ignorant women calling themselves nurses." This last danger is a real one, seeing that the British Nurses' Association has proposed to admit all its members to its register—that is, necessarily, to give a certificate to women who have no certificate of training nor any claim to one at the present time. We have always protested, and shall continue to protest in the interests of the really efficient nurses throughout the country, against any such unreasonable proceeding which is unnecessary, because the certificate of training can always be produced as evidence of competency; and wanton, in the fact that it would bring into the market as competitors to the really efficient a number of uncertificated women whose efficiency and character it must be difficult, if not impossible, to guarantee.

### Answers to Examination Questions.

What preparations should a nurse make (in a private house) for the following operations, and what should be her principal care during the first twenty-four hours after each operation? Ovariotomy, hare-lip, amputation of thigh, lithotomy?

FOR all operations of any importance at all, it is necessary for a nurse at a private house to see that there is a strong, narrow, moderately-high table, a good light in the room, and the room should be warmed and well ventilated. Several basins, plenty of hot and cold water, a tray, or a flat dish for instruments, whatever lotions the surgeon orders, strapping, perchloride of iron, sponges, absorbent wool, strong ammonia, two hot bottles, bandage, tow, green protective, a feeding cup, brandy, a catheter, a Higginson's syringe, and a hypodermic syringe, should be in the room. Needles, pins, thread, tape, scissors, waterproof sheeting, a box full of sawdust, and for an ovariotomy a piece of thinnish waterproof, with a circular hole in the centre, is generally provided. The edges of the hole are smeared with adhesive preparation. When the patient is unconscious, this is fixed over the abdomen; it keeps the patient dry and clean, saves needless exposure, shortens the washing, etc., after the operation. As regards antiseptic precautions, each doctor gives instructions as to what lotions he prefers. After the operation—when the patient is comfortably settled in bed—she must be kept flat on her back, and not allowed to sit up or make any undue movements. It is usual to give nothing for twenty-four hours, or longer, but a teaspoonful of iced milk now and then, or only a lump of ice to suck from time to time. Sickness is a danger—there is usually a little at first—and the nurse in this case must support or gently press on the abdomen in such a way as to prevent the wound being dragged by the straining; the catheter must be passed every four or six hours, as the doctor orders. It is most advisable to avoid having the bed wet, so that the draw sheet need not be changed or even drawn for twenty-four hours or so. This can generally be done by care, and in case of slight hæmorrhage or discharge from the vagina I have found a pad of absorbent wool very useful; it can be drawn away without disturbing the patient. Should hæmorrhage or discharge from the wound occur, the doctor should be sent for at once, and also if diarrhœa should set in, or if there should be a profuse discharge or much hæmorrhage from the vagina, or if the temperature should rise suddenly to, say, 103, and be accompanied by a very weak rapid pulse. Pulse temperature and respiration to be taken as the doctor directs.

### Everybody's Opinion.

*[Correspondence on all subjects is invited, but we cannot in any way be responsible for the opinions expressed by our correspondents. No communications can be entertained if the name and address of the correspondent is not given, or unless one side of the paper only be written on.]*

### ASYLUM ATTENDANTS.

A CHARGE NURSE writes: In THE HOSPITAL for December 7th there was a short article about asylum attendants. Banstead was the asylum mentioned. We were condemned for lack of "go" because we had sent no dolls for the competition. You were generous enough to find an excuse for us. You also stated we had a day off duty every two weeks, and ten days' leave annually. I beg to correct you. We are off duty three times during the month, two half days and one whole day. After our whole day we have fourteen days without intercession. We are allowed seven days' leave for the first five years. After that time we are entitled to ten days' leave. In last week's HOSPITAL there was a short article about long hours and over-worked nurses. You wished to know if hours were as long elsewhere. Our hours are from 6 a.m. till 8 p.m. all the year round.

### OVER-WORKED NURSES.

E. C. writes: Permit me to give you the names of two institutions where the day nurses work for fourteen hours every second day: St. Saviour's Infirmary, at East Dulwich, and The Miller Hospital, Greenwich-road, S.E. In both these institutions they promise day nurses one free day per month, but on the so-called "day off" keep the nurse at work from 7 to 10 a.m., and, besides this, give her a day when she would, in any case, be entitled to two or three hours in the afternoon. Accept a nurse's best thanks for your promise to draw public attention to a practice which you will, I believe, find to be almost universal.

Alix Roy writes: Since you invite comment, I can endorse emphatically every word that "A. E. S." has written with regard to long working hours and insufficient off-duty time in small provincial hospitals. The callous indifference evinced by hospital officials concerning the well-being of their nurses would raise a cry of indignation against accredited slave proprietors. It is natural that doctors and matrons should wish to get as much out of their nurses as is possible; but where their horses are in question they realise that it is bad policy to overwork the willing steed. For the comfort of the patients, and the credit of the institution, it would be well would they apply the same rule to staffs at their command. A certain minimum of off-duty daily, and a day "off" every six or eight weeks would make a world of difference. In the hospital of which I am an inmate, recreation is an unknown quantity. We are, however, supposed to have "off-duty" every other day, circumstances permitting. All work and no play has the inevitable result of making us a dull party. With the expansion of some branch of recreation's science we should be better nurses as well as happier women. As "A. E. S." has pointed out, we *cannot* strike. Our daily bread depends on the fulfilment of our labour, and the competition is very keen. With her, I have often thought it hard that while the grievances of working-men convulse a nation, the grievances of working-women should be practically set at naught. The notion of an eight-hours' Bill for nurses might well seem farcical to women who, like myself, are accustomed to be on foot twelve, fourteen, and sometimes fifteen hours daily.

### Lectures.

Mrs. R. Franks, late matron of Kensington Infirmary, and for many years sister at St. Thomas's Hospital, will give a course of lectures on Home Nursing, at St. George's Parish Room, Edge-street, Campden Hill, W., commencing on January 9th, at 2.30 p.m. Tickets, for the course of six lectures, price 10s. 6d.; a single lecture, 2s. 6d., to be had at the doors.

### Notes and Queries.

To Correspondents.—1. Questions may be written on post-cards. 2. Advertisements in disguise are inadmissible. 3. In answering a query please quote the number. 4. If a private answer is desired, a stamped addressed envelope must be forwarded.

Notice.—We must really ask our correspondents to be more particular in enclosing name and address. Constantly we receive queries or letters with no name attached.

### Answers.

(31) *Home Wanted.*—Try the Herbert Convalescent Home at Bournemouth, 12s. 6d. a week without a letter; or the Homœopathic Home, West Cliff, 7s. 6d. a week.

(33) *Home for Imbecile.*—It depends on what county the child belongs to. There is a home at Colchester for the Eastern Counties, and one at Exeter for the Western Counties. Then of course there is Earlswood by election.

*Guided.*—Apply to the Hospital for Women, Waterloo-bridge-road, S.W., or the Boston Hospital, Lincolnshire, or Grimsby District Hospital, or Lincoln County Hospital. The best plan is to answer the advertisements appearing in our pages.

### Death in our Ranks.

We regret to announce the death, on December 19th, of Mrs. M. O. Higginbotham, founder and superintendent of the Glasgow Sick Poor and Private Nursing Institution. Mrs. Higginbotham was the only daughter of Lady Whitworth, and her interest in the sick poor was such that she underwent a course of training in Mrs. McAlpine's Training Home in Glasgow, in order that she might devote the rest of her life to the services of those with whom she so sincerely sympathised. Under her direction the institution was formed, and grew till it reached its present powerful position. Mrs. Higginbotham's death is a severe loss to many of her friends.

### Vacancies.

The following vacancies are announced:

### Matrons.

Berkeley Hospital, £30.
Birmingham Lock Hospital.
Colchester Infectious Hospital, £35.
St. Alban's Hospital, £40.
Tyrone Infirmary, £40.

### Night Superintendent.

Chelsea Infirmary, £30.
Queen's Hospital, Birmingham, £30

### Nurses.

Barnstable Infirmary, £20.
Blackheath Institute.
Bournemouth Institute, £25.
Bournemouth Sanatorium, £25.
Brixton Institute.
Cambridge Home for Nurses.
Central London Throat and Ear Hospital, £20.
Cheltenham Nursing Institution.
Eastbourne Institution.
General Nursing Institute.
Hampstead Home Hospital, £20.
Hanwell Ophthalmic School, £25.
Highbury Institute.
Holborn Union (two night), £20.
Kent Nursing Institution, £30.
Kidderminster Infirmary, £25.
Leicester Institution, £25.
Loughborough Infirmary.
Liverpool Infectious Diseases Hospital (three), £25.
North-Eastern Hospital for Children, £20.
North-Western Fever Hospital, £30
Nottingham Nurses' Institute, £25
Nursing Institution, 96, 97, 98, Wimpole-street, London, W., Mr. Wilson has several vacancies for thoroughly-trained nurses.
Seamen's Hospital, S.E.
Sheffield Union (several).
South-Eastern College, Ramsgate, £30.
St. George's Infirmary.
St. Helena Home, N.W.
Swansea Nursing Institute, £25.
Ventnor Consumptive Hospital, £20

### District Nurses.

33, Hyde Park-gardens, W.
13, Lloyd-square, W.C.

### Probationers.

Boston Hospital.
Canterbury Nurses' Institute.
Hull Royal Infirmary.
Newport and County Infirmary.
Royal Surrey Hospital.

### Amusements and Relaxation.

### Third Quarterly Word Competition Commenced October 5th, 1889, ends December 28th, 1889.

Three Prizes of 15s., 10s., 5s., will be given for the largest number of words derived from the words set for dissection.

Proper names, abbreviations, foreign words, words of less than four letters, and repetitions are barred; plurals, and past and present participles of verbs, are allowed. Nuttall's *Standard* dictionary only to be used.

N.B.—Word dissections must be sent in WEEKLY, arranged alphabetically, with correct total affixed.

The word for dissection for this, the *Thirteenth* week of the quarter, being "BOARS HEAD."

### Results of Eleventh Week.

| Names. | Dec. 19th. | Totals. | Names. | Dec. 19th. | Totals. |
|---|---|---|---|---|---|
| Winifred | — | 672 | Electrician | 142 | 1481 |
| Adelaide | — | 646 | H. C. | — | 562 |
| Nettie Vance | — | 757 | Reldas | 147 | 1543 |
| F. W. P. H. | 155 | 1543 | Rover | — | 776 |
| Amos | — | 596 | Qu'appelle | 143 | 1274 |
| Reyot | — | 369 | Beryl | — | 188 |
| Ellice | 155 | 1569 | Lightowlers | 151 | 1591 |
| A. S. K. | — | 275 | Jenny Wren | 141 | 1146 |
| Neptune | — | 1314 | Hercules | — | 12 |
| Ecila | 155 | 1627 | Night Watch | — | 346 |
| Tinie | — | 17 | Oakenrod | — | 1170 |
| Amicus | — | 946 | Embryo | — | 385 |
| Juvenis | — | 539 | Gypsye | — | 967 |
| Jumps | 157 | 1625 | Leo | 145 | 844 |
| Sister | — | 438 | Pearl | — | 299 |
| M. W. | 155 | 1567 | Africando | — | 171 |
| Esperance | 155 | 1566 | Lady Clara | 133 | 133 |
| Sunshine | 143 | 1338 | | | |

### Notice to Correspondents.

N.B.—All letters referring to this page which do not arrive at 139 and 140, Salisbury-court, London, E.C., by the *first post on Thursdays*, and are not addressed PRIZE EDITOR, will in future be disqualified and disregarded.

